# MicroRNAs in Spinal Cord Injury: Molecular and Translational Insights

**DOI:** 10.1002/brb3.71617

**Published:** 2026-07-28

**Authors:** Seyyedeh Fahimeh Talebi, Hossein Kalarestaghi, Ehsan Sheikh Hesabi, Saeed Vafaei‐Nezhad

**Affiliations:** ^1^ Immunology Research Center Tabriz University of Medical Sciences Tabriz Iran; ^2^ Research Laboratory for Embryology and Stem Cell, Department of Anatomical Sciences, School of Medicine Ardabil University of Medical Sciences Ardabil Iran; ^3^ School of Medicine Ardabil University of Medical Sciences Ardabil Iran; ^4^ Department of Anatomy, School of Medicine, Medical Toxicology and Drug Abuse Research Center Birjand University of Medical Sciences Birjand Iran

**Keywords:** apoptosis, inflammation, microRNA, spinal cord injury

## Abstract

**Background:**

Spinal cord injury (SCI) is characterized by complex molecular and cellular disturbances that contribute to progressive tissue damage and neurological dysfunction. Among the regulatory mechanisms implicated, microRNAs (miRNAs), which are small noncoding RNAs that regulate gene expression posttranscriptionally, have emerged as central components of several injury‐related pathways. This review synthesizes current knowledge regarding the regulatory functions of miRNAs and evaluates their potential as therapeutic targets.

**Method:**

Recent experimental and preclinical studies were analyzed to identify key miRNAs associated with injury‐induced molecular responses and to assess advances in miRNA‐based therapeutic strategies, including the use of miRNA mimics, inhibitors, and delivery systems.

**Results:**

Several miRNAs, including miR‐21, miR‐223, miR‐124, and miR‐219, can regulate essential biological processes such as apoptosis, neuroinflammation, oxidative stress, glial activation, and remyelination. miR‐21 and miR‐223 exhibited context‐dependent roles in neuroinflammation, apoptosis, and vascular repair, while miR‐124 could modulate microglial activity and miR‐219 facilitates oligodendrocyte differentiation and myelin restoration. Experimental therapeutic approaches employing viral vectors, nanoparticles, stem cell–based delivery, and exosome systems have resulted in enhanced tissue preservation, angiogenesis, and functional outcomes in preclinical models.

**Conclusion:**

miRNAs serve as critical molecular regulators and represent promising therapeutic targets. Nevertheless, clinical translation is constrained by challenges such as delivery barriers, off‐target effects, and the complexity of miRNA‐mediated regulatory networks. Advances in delivery technologies and research focused on precise miRNA regulation may support the development of effective neuroprotective and regenerative therapies.

## Graphical abstract

MicroRNAs (miRNAs) modulate secondary injury cascades following spinal cord injury (SCI), including inflammation, oxidative stress, apoptosis, and demyelination. Key miRNAs such as miR‐21, miR‐124, miR‐219, and miR‐223 exert neuroprotective effects by regulating astrocytic response, axon regeneration, and remyelination. These actions collectively promote functional recovery and reduce neurological impairments after SCI.

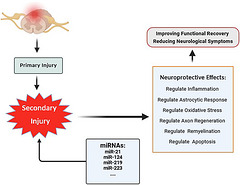

## 1 Overview of Spinal Cord Injury (SCI)

Traumatic SCI is a leading cause of mortality and morbidity worldwide. Recent reports indicate that the incidence of SCI is increasing annually, with epidemiological characteristics varying across regions. The global prevalence of SCI is estimated at approximately 20.6 million, with an additional 250,000–500,000 new cases occurring each year (McDonald and Sadowsky 2002). SCI is a life‐threatening neurological disorder characterized by autonomic, sensory, and motor impairments, which result in reduced life expectancy. The extent of this reduction depends on the patient's age at the time of injury and the severity of the SCI (Patek and Stewart 2026).

Multiple studies have demonstrated that microRNAs (miRNAs) play a critical role in modulating the pathophysiology of SCI. MiRNAs can either mitigate or worsen secondary injury by regulating key biological processes such as apoptosis, inflammation, oxidative stress, and demyelination or remyelination. According to the latest release of the miRBase database (version 22.1), 48,860 mature miRNAs derived from 38,589 precursor miRNAs (pre‐miRNAs) have been annotated across 271 species. In the human genome, 2654 mature miRNAs originate from 1917 pre‐miRNAs (Kozomara et al. 2019). These evolutionarily conserved noncoding RNAs regulate a significant proportion of protein‐coding genes and are essential in various physiological and pathological processes. Furthermore, miRNAs are estimated to regulate more than 60% of human protein‐coding genes, highlighting their central role in posttranscriptional gene regulation (Friedman et al. 2009; Bartel 2018).

This review aims to examine current evidence on the role of miRNAs in SCI, with a particular focus on key post‐injury molecular mechanisms.

## 2 Pathophysiology of SCI

**FIGURE 1:**
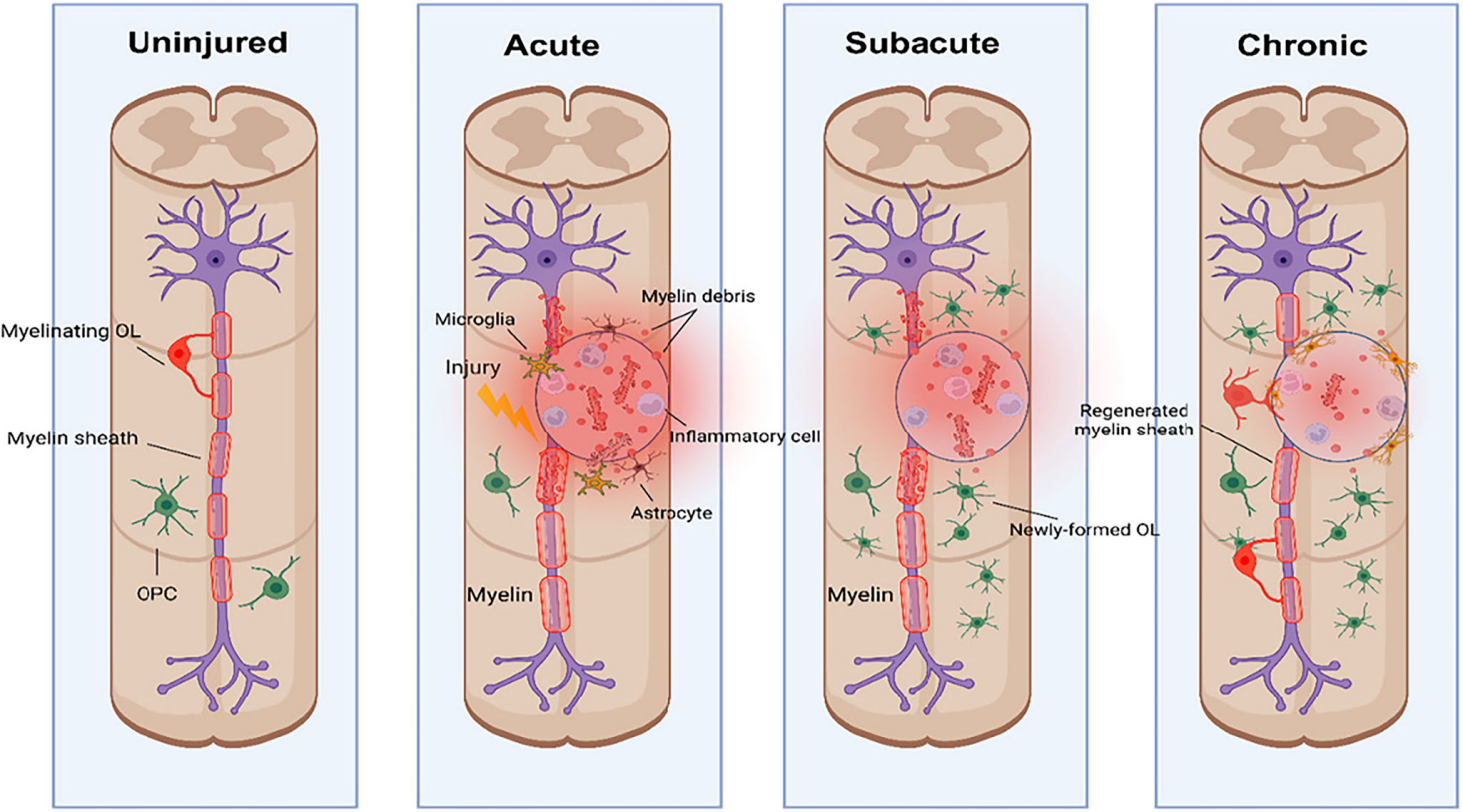
Pathophysiology of spinal cord injury. The primary injury causes immediate mechanical damage, while the secondary phase triggers cascades of ischemia, oxidative stress, inflammation, apoptosis, demyelination, and glial scar formation across acute, subacute, and chronic stages, leading to progressive tissue loss and dysfunction.

Both nontraumatic and traumatic etiologies (Figure 1) contribute to the incidence of SCI. Congenital abnormalities, infections, and tumors are the most recognized causes of nontraumatic SCI, whereas traffic accidents, falls, and sports‐related trauma are the primary causes of traumatic SCI (Vafaei‐Nezhad et al. 2021). Clinically, the extent of injury is a critical factor, as it may lead to cardiovascular and respiratory complications, as well as impaired bowel and bladder control.

From a pathophysiological perspective, SCI progresses through two sequential phases: primary and secondary injury (Figure 1). The primary injury results from direct mechanical trauma to the spinal cord, such as vertebral compression, hyperextension, distraction forces, or laceration by bone fragments or external objects, leading to immediate structural damage to neural tissues (Hachem and Fehlings 2021). Hemorrhage, resulting from vascular rupture within the spinal cord or localized bleeding at the injury site, is a major hallmark of this phase. Vascular disruption induces ischemia by depriving spinal cord cells of essential nutrients and oxygen, causing significant cellular damage. Furthermore, the duration of spinal cord compression and the magnitude of the initial insult determine the overall severity of SCI. Because the primary injury is irreversible, a comprehensive understanding of its underlying mechanisms is essential for developing therapeutic strategies aimed at limiting subsequent secondary damage (Anjum et al. 2020; Hasan et al. 2025). Moreover, secondary injury may begin within minutes of the primary insult and can persist for several months, further exacerbating spinal cord damage. A complex cascade of cellular, molecular, and biochemical mechanisms impedes spinal cord recovery during this period. The secondary injury process comprises three stages: acute, subacute, and chronic phases. These stages are characterized by features such as vascular dysfunction, inflammatory responses, apoptosis, axonal degeneration, demyelination, and glial scar formation (Migliorini et al. 2025).

Primary injury initiates cellular processes, such as oxidative stress, and systemic responses, including immune activation, which contribute to secondary injury. Early therapeutic intervention can reduce neurological damage and enhance long‐term functional recovery. Surgical approaches performed within 8–24 h after injury have been shown to limit secondary damage by restoring vascular perfusion and alleviating mechanical pressure on the spinal cord (Lima et al. 2022). Additionally, multiple studies have demonstrated that miRNAs regulate key genes and molecular pathways implicated in the pathogenesis of secondary SCI (Sintakova and Romanyuk 2024).

## 3 MiRNAs: Definition, Function, and Biogenesis

MiRNAs are small, highly conserved, single‐stranded noncoding RNAs, approximately 22 nucleotides in length, that regulate gene expression at the posttranscriptional level. The primary binding site for miRNAs is the 3′ untranslated region (3′ UTR) of target mRNAs, leading to either mRNA degradation or inhibition of protein translation. By modulating essential biological processes such as cell death, proliferation, and metabolism, miRNAs are integral to the maintenance of normal cellular function. Consequently, dysregulation of miRNA function or expression can contribute to the onset and progression of various diseases (Shang et al. 2023). The biogenesis of miRNAs is a tightly regulated, multistep process. MiRNA genes are transcribed by RNA polymerase II to generate primary miRNA transcripts (pri‐miRNAs). Within the nucleus, these pri‐miRNAs are processed by the Drosha–DGCR8 complex into pre‐miRNAs. The pre‐miRNAs are subsequently exported to the cytoplasm via Exportin‐5 and further cleaved by the RNase III enzyme DICER to produce an approximately 22‐nucleotide miRNA duplex. One strand of this duplex is incorporated into the Argonaute‐containing RNA‐induced silencing complex (RISC), which directs the mature miRNA to its target mRNA. This mechanism results in gene silencing through translational inhibition or mRNA degradation (Shang et al. 2023).

## 4 Potential Roles of MiRNA in SCI

### 4.1 MiRNAs Regulate Oxidative Stress After SCI

Dysregulated miRNA expression contributes to oxidative stress–related diseases by modulating key signaling pathways, such as Nrf2, SIRT1, and NF‐κB. Reactive oxygen species (ROS) influence miRNA expression by upregulating or downregulating specific miRNAs, thereby affecting gene expression and cellular responses (Yin et al. 2024). Experimental evidence indicates that reducing oxidative stress mitigates SCI‐induced damage in animal models (Choi et al. 2012; Jia et al. 2012). NADPH oxidase 4 (NOX4), a primary neuronal source of ROS, plays a critical role in the regulation of oxidative stress and inflammation [16]. SCI leads to increased NOX expression, which further amplifies oxidative damage. Inhibition of NOX4 suppresses inflammasome activation in macrophages, underscoring its therapeutic potential (Mursal et al. 2025).

Overexpression of miR‐99a enhances cell viability, downregulates proapoptotic markers Bax and cleaved caspase‐3, and upregulates the antiapoptotic protein Bcl‐2. miR‐99a also reduces ROS accumulation and inflammatory cytokines, including IL‐6, IL‐1β, and TNF‐α, in lipopolysaccharide (LPS)‐treated PC‐12 cells by directly targeting NOX4 (Wang et al. 2022). In SCI models, miR‐99a improves motor function and reduces inflammation, apoptosis, and oxidative stress [18]. Furthermore, miR‐137 alleviates SCI‐induced damage by targeting Neuronal Differentiation 4 (NeuroD4) and suppressing oxidative stress and inflammation (Dai et al. 2018). Downregulation of miR‐96 following SCI increases apoptosis by upregulating proapoptotic target proteins (Table 1) (Almurshidi et al. 2019). In contrast, restoration of miR‐96 suppresses microglial activation and decreases inflammatory cytokines, including TNF‐α and IL‐1β, thereby promoting recovery (Almurshidi et al. 2019). miR‐96‐5p also regulates cysteine transporters, such as excitatory amino acid transporter 3 (EAAT3), which are essential for glutathione synthesis and antioxidant defense (Almurshidi et al. 2019; Kinoshita et al. 2021).

**TABLE 1:** MiRNAs and apoptosis.

MiRNA	Expression after SCI	Major target(s)	Biological significance	Ref
Let‐7	↑	RAS, MYC, Bcl‐2	Promoted apoptosis via Ras/Bcl‐2 signaling.	(Almurshidi et al. 2019; Liu et al. 2010)
miR‐15b	—	Bcl‐2	Promoted apoptosis by targeting Bcl‐2.	(Almurshidi et al. 2019; Liu et al. 2010)
miR‐16	↑	Bcl‐2	Promoted apoptosis by targeting Bcl‐2.	(Almurshidi et al. 2019; Cimmino et al. 2005; Liu et al. 2010)
miR‐20a	↑	Mcl‐1, NEUROG1	Promoted neuronal apoptosis by targeting Mcl‐1; NEUROG1 suppression may impair neuronal regeneration.	(Liu et al. 2015; Martirosyan et al. 2016)
miR‐21	↑	PTEN, PDCD4	Suppressed apoptosis via PTEN/PDCD4 inhibition.	(Malvandi et al. 2022; Almurshidi et al. 2019; Liu et al. 2010)
miR‐29b	↓	Bad, Bim, Noxa, PUMA (BH3‐only proteins)	Suppressed apoptosis by inhibiting BH3‐only proteins.	(Martirosyan et al. 2016; Liu et al. 2015)
miR‐96	↓	FOXO1, FOXO3a	Suppressed apoptosis by targeting FOXO1/FOXO3a.	(Almurshidi et al. 2019; Guo et al. 2012)
miR‐124	—	BIM (BCL2L11)	Suppresses apoptosis by targeting BIM and is associated with improved recovery after SCI.	(Zhang et al. 2022)
miR‐200c	↑	FAP‐1	Promotes ROS‐induced apoptosis by targeting FAP‐1.	(Yu et al. 2015)
miR‐486	↑	NEUROD6	Promoted oxidative stress and apoptosis via NeuroD6 suppression.	(Jee et al. 2012; Martirosyan et al. 2016)

miR‐21 primarily contributes to ROS‐mediated apoptosis during oxidative stress. Silencing miR‐21 reduces ROS‐induced neuronal death in spinal cord neurons, underscoring its critical role in oxidative stress–related cellular injury (Table 1) (Martirosyan et al. 2016; Hasan et al. 2025). miR‐486 targets NEUROD6, which modulates heat shock proteins and neuronal survival (Jee et al. 2012). Silencing miR‐486 increases NEUROD6 expression, thereby enhancing ROS clearance and reducing pro‐inflammatory mediators. NEUROG1, which is highly expressed in neural progenitor cells, regulates neuronal differentiation during embryonic development (Jee et al. 2012). miR‐20a is upregulated after SCI and targets NEUROG1, inhibiting neuronal regrowth and promoting motor neuron degeneration and apoptosis (Liu et al. 2015). Inhibition of miR‐20a may enhance neuronal regeneration and functional recovery. Hence, miR‐29b exerts antiapoptotic effects by suppressing BH3‐only genes during ischemia, suggesting that combined modulation of miR‐20a and miR‐29b may represent a potential therapeutic strategy (Liu et al. 2015; Ebada et al. 2023).

### 4.2 MiRNAs Regulate Apoptosis After SCI

Apoptosis plays a significant role in inflammation, excitotoxicity, and progressive neuronal loss associated with secondary damage following SCI. It also affects the tissue surrounding the lesion site, as apoptotic cells release inflammatory cytokines and free radicals. This process intensifies secondary injury, activates microglia, and disrupts homeostasis. Preventing apoptosis remains a major challenge in SCI treatment because spinal cord neurons possess extremely limited regenerative capacity (Zhang et al. 2012). Oligodendrocytes (OLs), which are responsible for axonal myelination, are particularly vulnerable, undergoing both necrotic and apoptotic death after SCI. Loss of OLs through apoptosis can result in widespread demyelination and impaired axonal conduction (Kim et al. 2003). Fas receptor‐mediated apoptosis may be triggered by demyelination, leading to activation of caspase‐8 and caspase‐3 and further OL loss. Apoptosis after SCI occurs via two principal pathways: the extrinsic (death receptor–mediated) and intrinsic (mitochondrial) pathways. The extrinsic pathway involves activation of TNFR, Fas, and TRAIL receptors, which subsequently activate caspase‐8, caspase‐10, and caspase‐3. Mitochondrial damage and cytochrome c release initiate the intrinsic pathway, resulting in apoptosome formation with Apaf‐1 and caspase‐9. Both pathways ultimately converge on caspase‐3 (Savitskaya and Onishchenko 2015). Additional factors, including ROS and bioactive molecules, further stimulate apoptotic signaling (Savitskaya and Onishchenko 2015).

MiRNAs serve as critical regulators of apoptosis in SCI by targeting genes involved in cell survival and death. Bioinformatics analyses indicate that miR‐20a and miR‐29b modulate MCL‐1 and BH3‐only genes (Table 1) (Liu et al. 2015). Experimental evidence demonstrates that administration of a miR‐20a mimic or a miR‐29b inhibitor promotes neuronal apoptosis, whereas co‐injection of both agents inhibits apoptosis in the injured spinal cord. Collectively, these findings suggest that miR‐20a and miR‐29b contribute to neuronal loss following SCI by downregulating the antiapoptotic protein Mcl‐1 and upregulating proapoptotic BH3‐only proteins (Liu et al. 2015). Overexpression of miR‐21, which is observed within 24 h after SCI and remains elevated for several weeks, reduces neuroinflammation by suppressing pro‐inflammatory cytokines and enhancing anti‐inflammatory responses (Lv et al. 2020). Elevated miR‐21 levels also reinforce the blood–spinal cord barrier (BSCB), regulate angiogenesis, and influence glial scar formation (Malvandi et al. 2022). By targeting proapoptotic genes such as FasL, PTEN, and PDCD4, miR‐21 confers protection against cell death. Inhibition of miR‐21 with antagomir‐21 impairs recovery, increases apoptosis, and enlarges lesion size. miR‐21 limits death receptor signaling by targeting FasL and, through PTEN inhibition, enhances Akt‐mediated cell survival and neurogenesis (Hu et al. 2013). miR‐96 also exhibits antiapoptotic properties (Almurshidi et al. 2019). Upregulation of miR‐96 suppresses PDCD4 and caspase‐9, thereby protecting against mitochondrial‐mediated apoptosis. miR‐96‐5p targets the CASP9 3′ UTR and regulates FOXO1, modulating intrinsic apoptotic signaling (Table 1) (Almurshidi et al. 2019). miR‐96 activates the Akt pathway by suppressing PTEN and PDCD4, which promotes neuronal survival and functional recovery (Almurshidi et al. 2019). miR‐124 is also essential due to its negative regulation of BIM (Zhang et al. 2022). Suppression of miR‐124 increases BIM expression and apoptosis, whereas overexpression reduces apoptosis. A polymorphism (rs531564) may decrease miR‐124 levels and result in prolonged functional recovery after SCI (Zhang et al. 2022).

In contrast to apoptosis‐suppressing miRNAs, certain miRNAs promote apoptosis. Let‐7a acts as a proapoptotic factor by targeting antiapoptotic genes, including RAS and MYC (Table 1) (Liu et al. 2010; Johnson et al. 2005). miR‐223, which is overexpressed following SCI, is implicated in the regulation of neuronal apoptosis, inflammation, and vascular repair (Liu et al. 2015). Inhibition of miR‐223 enhances hindlimb motor function and reduces neuronal death, which correlates with decreased Bax and caspase‐3 expression and increased Bcl‐2 expression. miR‐223 also modulates neuroinflammation and glutamate receptor signaling, influencing NMDA and AMPA receptor subunits (Liu et al. 2015; Pinchi et al. 2019; Liu et al. 2022). miR‐200c, another proapoptotic miRNA, is markedly upregulated after SCI and induces apoptosis in BV‐2 microglial cells through increased ROS production. miR‐200c activates the Fas pathway by downregulating FAP‐1 and shifts Bcl‐2 family protein expression toward apoptosis. It reduces Bcl‐2 levels while increasing Bax and Bim expression. Suppression of miR‐200c reverses these effects, highlighting its therapeutic potential (Table 1) (Yu et al. 2015).

### 4.3 MiRNAs Regulate Inflammation After SCI

Inflammation constitutes a significant aspect of the secondary phase of SCI, commencing within minutes after trauma and potentially persisting for years. This response exhibits both beneficial and detrimental effects: It facilitates the clearance of necrotic debris and restricts toxic byproducts that impede axonal regeneration, yet excessive or prolonged activation intensifies neural damage. Key inflammatory cytokines, such as TNF‐α, IL‐1β, and IL‐6, enhance immune cell infiltration and promote neuroinflammation (Hellenbrand et al. 2021). The inflammatory cascade following SCI is categorized into four phases: immediate, acute, intermediate, and chronic. Immediately after injury, disruption of the BSCB activates resident microglia and permits rapid infiltration of neutrophils, which may result in oxidative stress, excitotoxicity, and neuronal apoptosis. During the acute phase, macrophages and T lymphocytes accumulate at the injury site, contributing to both inflammatory damage and early tissue repair. Microglial activation peaks in the initial days and plays a critical role in regulating immune cell recruitment. Although neutrophils assist in removing damaged tissue, their prolonged presence can exacerbate secondary injury (Hellenbrand et al. 2021). Cytokines released by microglia, astrocytes, and macrophages coordinate the inflammatory response. Pro‐inflammatory mediators, such as IL‐1β and IL‐6, induce neuronal damage and apoptosis, whereas IL‐10 and TGF‐β promote tissue repair and neuronal survival. ROS generated at the injury site further increase oxidative stress and cellular injury. Although certain cytokines and glial responses contribute to neuronal damage, they are also essential for recovery and regeneration, underscoring the complexity of the inflammatory response. Consequently, many therapeutic strategies aim to selectively modulate, rather than broadly suppress, inflammatory pathways to enhance recovery (Hellenbrand et al. 2021).

miR‐126 serves as a principal endothelial regulator, promoting angiogenesis and attenuating vascular inflammation by targeting SPRED1, PIK3R2, and VCAM1. Following SCI, miR‐126 expression declines, prompting interest in therapeutic replacement strategies. Experimental upregulation of miR‐126 has been shown to enhance angiogenesis, reduce leukocyte infiltration, downregulate its target genes, and improve functional recovery in a dose‐dependent manner (Table 2) (Hu et al. 2015). miR‐223‐5p functions as an inflammatory regulator; its suppression inhibits pro‐inflammatory M1 microglial activation and promotes anti‐inflammatory M2 polarization. Reduced miR‐223‐5p expression leads to decreased IL‐1β, IL‐6, and TNF‐α levels, while increasing neuregulin 1 (NRG1) within 3 days post‐injury. At later stages, miR‐223‐5p suppression lowers caspase‐3 and glial fibrillary acidic protein (GFAP) expression, indicating reduced apoptosis and astrocytic activation (Guan et al. 2019). Hypoxia‐induced miR‐210 is a critical regulator of angiogenesis and tissue regeneration. Transcriptomic analyses in ischemic models demonstrate that miR‐210 modulates vascular repair and inflammatory signaling. Studies utilizing miR‐210 transgenic mice or anti‐miR‐210 inhibitors reveal that miR‐210 overexpression significantly reduces macrophage accumulation and inflammatory cell density in ischemic tissues, supporting its anti‐inflammatory potential (Zaccagnini et al. 2021). miR‐124 is essential for neuronal differentiation and microglial regulation. In microglia, miR‐124 promotes polarization toward the anti‐inflammatory M2 phenotype and reduces TNF‐α, MHC‐II, and ROS production. It also maintains microglial quiescence and modulates the activation of monocytes and macrophages (Zhao et al. 2021). miR‐146a demonstrates strong anti‐inflammatory properties. In vitro SCI models indicate that overexpression of miR‐146a significantly reduces TNF‐α, IL‐1β, and IL‐6 levels, primarily through inhibition of Toll‐like receptor 4 (TLR4) signaling, a central pathway in post‐injury inflammation (Tan et al. 2018). miR‐21 is another key anti‐inflammatory miRNA in the nervous system. Suppression of miR‐21 increases IL‐1β, IL‐6, TNF‐α, and receptor activator of nuclear factor‐κB ligand (RANKL), thereby exacerbating inflammation. Conversely, overexpression of miR‐21 reduces these cytokines and enhances IL‐10 production. miR‐21 mediates these effects by inhibiting NF‐κB, TLR/MyD88/NF‐κB, and JAK‐STAT pathways, thereby limiting secondary injury after SCI (Malvandi et al. 2022). In contrast, miR‐136‐5p acts as a pro‐inflammatory regulator. Upregulation of miR‐136‐5p increases IL‐1β, IL‐6, and TNF‐α, suppresses the anti‐inflammatory protein A20, and activates NF‐κB signaling. Silencing miR‐136‐5p protects spinal cord tissue by restoring A20 levels and reducing inflammatory signaling through the NF‐κB/A20 pathway (Table 2) (Deng et al. 2018). Finally, miR‐140 contributes to post‐injury inflammatory pathology by regulating astrocyte proliferation. By targeting brain‐derived neurotrophic factor (BDNF) via PI3K/Akt signaling, miR‐140 reduces BDNF, IL‐6, and TGF‐α expression in injury models, suggesting that the miR‐140/BDNF axis may serve as a target for modulating astrocyte‐driven inflammation (Tu et al. 2017).

**TABLE 2:** Inflammation‐associated miRNAs in SCI.

MiRNA	Expression after SCI	Major target(s)	Biological significance	Ref
miR‐99a	↓	NOX4	Suppressed inflammation, oxidative stress, and apoptosis.	(Wang et al. 2022)
miR‐126	↓	SPRED1, PIK3R2, VCAM1	Reduced inflammation and promoted angiogenesis.	(Hu et al. 2015)
miR‐223	↑	IL‐1β, IL‐6, TNF‐α, NRG‐1	Promoted neuroinflammation and impaired recovery.	(Guan et al. 2019)
miR‐223	Not determined (no SCI model)	RhoB	Preserved endothelial barrier integrity by suppressing apoptosis.	(Liu et al. 2022)
miR‐223	↑	GluR2	Promoted apoptosis and inhibits angiogenesis.	(Liu et al. 2015)
miR‐223	↑	NLRP3, GluR2	Promoted inflammation and apoptosis.	(Nakanishi et al. 2010; Pinchi et al. 2019)

### 4.4 MiRNAs Regulate Astrocytic Response After SCI

Astrocytes are glial cells that maintain homeostasis, support synaptic plasticity, and provide neuroprotection. After SCI, astrocytes exhibit both beneficial and detrimental effects (Gradisnik and Velnar 2023). In response to SCI, astrocytes migrate toward the lesion epicenter and initially facilitate tissue repair. During the early stages, reactive astrocytes clear excess glutamate, thereby reducing excitotoxic damage (Renault‐Mihara et al. 2008). In the chronic phase of SCI, astrocytes contribute to glial scar formation, a process initiated by injury‐induced signaling that progresses over several months. As the scar matures, astrocytes and fibroblasts deposit extracellular matrix (ECM) components such as chondroitin sulfate proteoglycans (CSPGs), fibronectin, and collagen, which stabilize the lesion site (Tran et al. 2022; Iaci et al. 2007). The mature glial scar comprises astrocytes, fibroblasts, neural stem/progenitor cells, microglia, macrophages, and infiltrating immune cells, collectively forming a dense physical and biochemical barrier. Among the inhibitory factors, CSPGs represent significant impediments to axonal regeneration (Yang et al. 2024). While glial scars restrict long‐term regeneration, they provide acute protection by containing inflammation and limiting lesion expansion. Therefore, astrocytes initially promote repair but subsequently adopt scar‐forming phenotypes that inhibit axonal growth. Therapeutic strategies that modulate these stages may enhance CNS regeneration following SCI.

miR‐21 functions as a key regulator of astrocyte proliferation, secretion, and apoptosis through the bone morphogenetic protein (BMP), JAK/STAT, and TGF‐β/PI3K/Akt/mTOR signaling pathways. Overexpression of miR‐21 attenuates astrocyte hypertrophy, while its inhibition intensifies this response. Additionally, miR‐21 mediates the transition from hypertrophy to hyperplasia by suppressing GFAP and vimentin expression under the control of BMP receptors. Activation of BMPR1a increases miR‐21 levels and decreases GFAP expression, thereby counteracting BMPR1b‐mediated pro‐scar signaling during chronic SCI (Almurshidi et al. 2019; Malvandi et al. 2022).

Deletion of DICER1, which is essential for miRNA maturation, leads to reduced astrocyte proliferation following injury. Administration of miR‐17‐5p mimics restores proliferation in Dicer1‐deficient astrocytes, while inhibition of miR‐17‐5p suppresses LPS‐induced proliferation, likely via the JAK/STAT3 signaling pathway (Fan et al. 2023; Hong et al. 2014). In addition, the lncRNA H19 functions as a competing endogenous RNA, counteracting miR‐1‐3p–mediated inhibition of CCL2. The H19/miR‐1‐3p axis thus regulates astrocyte proliferation and glial scar formation (Table 3) (Fan et al. 2023; Li et al. 2020). Overexpression of miR‐146a and miR‐147b reduces IL‐6 and cyclooxygenase‐2 (COX‐2) expression in astrocytes, thereby attenuating inflammation and limiting proliferation (Bai et al. 2021; Iyer et al. 2012). miR‐145, which is normally expressed in spinal neurons and astrocytes, is downregulated after SCI and further suppressed by LPS‐induced inflammation. Overexpression of miR‐145 decreases astrocyte density, hypertrophy, proliferation, and migration. miR‐145‐5p also inhibits proliferation by suppressing SMAD3, a key promoter of glial scar formation (Table 3) (Fan et al. 2023; Wang et al. 2015). Furthermore, anti‐miR‐125b reduces IL‐6‐stimulated astrocyte proliferation by upregulating CDKN2A, indicating potential therapeutic applications for limiting glial hyperplasia (Almurshidi et al. 2019; Li et al. 2016).

**TABLE 3:** MicroRNAs regulating astrocytic responses after SCI.

MiRNA	Expression after SCI	Major target(s)	Biological significance	Ref
miR‐1‐3p	↓ In LPS‐stimulated astrocytes	CCL2; H19/miR‐1‐3p axis	Suppressed astrocyte proliferation via CCL2 inhibition.	(Fan et al. 2023; Li et al. 2020)
miR‐21	Context‐dependent	PTEN, PDCD4, FasL, Smad7, GFAP, vimentin; PI3K/Akt pathway	Exerts phase‐dependent effects, supporting early neuroprotection while contributing to later glial scar remodeling.	(Hasan et al. 2025; Almurshidi et al. 2019; Malvandi et al. 2022)
miR‐136‐5p	↑ After SCI	A20/TNFAIP3; NF‐κB pathway	Promoted neuroinflammation via A20/NF‐κB signaling.	(Fan et al. 2023; He et al. 2017; Deng et al. 2018)
miR‐140	Not reported; experimentally overexpressed	BDNF; PI3K/Akt pathway	Suppressed astrocyte proliferation and inflammation.	(Fan et al. 2023; Tu et al. 2017)
miR‐145	↓ After SCI	GFAP, c‐Myc	Suppressed astrogliosis via GFAP/c‐Myc inhibition.	(Wang et al. 2015)
miR‐145‐5p	↓ After SCI	SMAD3	Suppressed astrocyte proliferation via SMAD3.	(Fan et al. 2023; Ye et al. 2022)
miR‐146a/miR‐147b	Not SCI study; ↑ in temporal lobe epilepsy with hippocampal sclerosis	IL‐6, COX‐2	Suppressed inflammation and astrocyte proliferation.	(Bai et al. 2021; van Scheppingen et al. 2018)

### 4.5 MiRNAs Regulate Axon Regeneration and Remyelination After SCI

OLs are the central nervous system's myelin‐producing glial cells that generate myelin sheaths around axons, thereby facilitating efficient saltatory conduction and providing metabolic support. After SCI, OLs are highly susceptible to secondary damage resulting from oxidative stress, excitotoxicity, and inflammatory processes. The degeneration of these cells leads to demyelination, impaired axonal function, and persistent neurological deficits. Although OL progenitor cells (OPCs) remain in the injured spinal cord and can initiate some spontaneous remyelination, this endogenous repair mechanism is constrained by the adverse post‐injury microenvironment. Major extrinsic barriers include myelin‐associated inhibitors (MAIs) and growth‐inhibitory molecules originating from the glial scar, which are produced by OLs, astrocytes, microglia, and fibroblasts (Shokraneh 2019). Key inhibitory factors include myelin‐associated glycoprotein (MAG), Nogo‐A, OL myelin glycoprotein (OMgp), and CSPGs. These molecules substantially impede axonal regeneration and remyelination.

**FIGURE 2:**
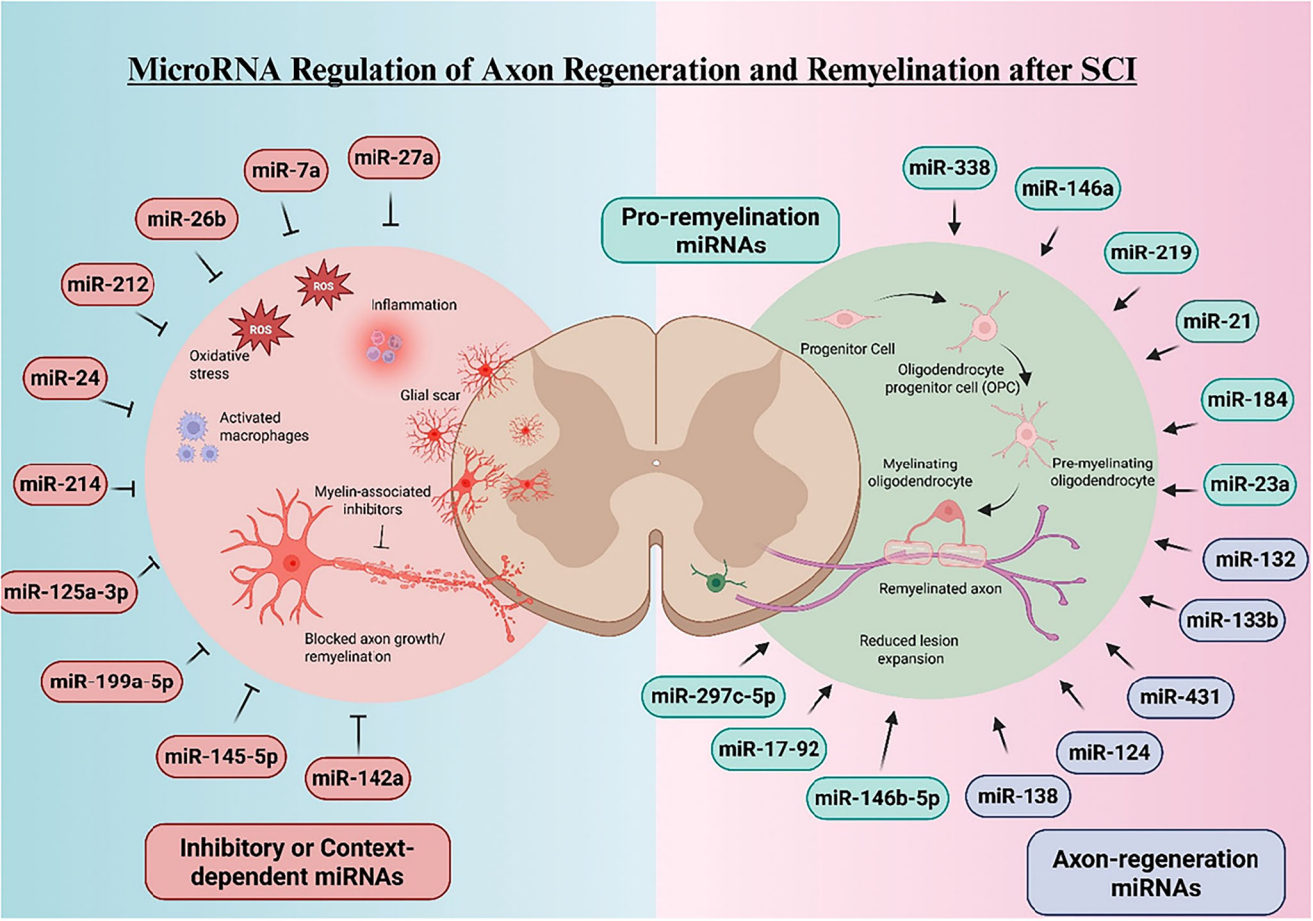
Schematic overview of miR‐mediated regulation of axon regeneration and remyelination following SCI. Pro‐regenerative miRNAs promote oligodendrocyte differentiation, myelin repair, axonal growth, and functional recovery, whereas inhibitory or context‐dependent miRNAs contribute to inflammation, oxidative stress, glial scar formation, and impaired regeneration. Together, these miRNAs influence the balance between tissue repair and persistent neurological dysfunction after SCI.

MiRNAs are recognized as critical regulators of OL lineage progression and regenerative processes. The RNase III enzyme Dicer1, required for miRNA maturation, is indispensable for CNS myelination and OPC differentiation. Dicer1 influences transcription factors such as Sox and Zfp and modulates key developmental pathways, including Notch and Wnt. Genetic ablation of Dicer1 results in increased OPC proliferation but impairs myelination, demonstrating that miRNAs are necessary for the transition from proliferation to differentiation (Lee and Zheng 2012; Qiu et al. 2024; Zawadzka et al. 2022). miR‐21 serves as a regulator of neural stem cell (NSC) fate and glial responses following SCI. Overexpression of miR‐21 promotes NSC differentiation toward the OL lineage and facilitates remyelination. Mechanistically, miR‐21 inhibits Smad7 and activates TGF‐β/Smad2 signaling, a process associated with glial scar formation and diminished remyelination. Inhibition of miR‐21 can reverse these effects. miR‐21 also enhances NSC proliferation by upregulating cyclin D1 through activation of the PI3K/AKT and AKT/GSK‐3β pathways, whereas knockdown of miR‐21 disrupts these signaling cascades. Furthermore, miR‐21 modulates Wnt/β‐catenin signaling, thereby promoting neuronal differentiation and restricting astrocytic lineage commitment (Figure 2) (Hasan et al. 2025). In astrocytes, miR‐21 overexpression suppresses beneficial hypertrophic responses, while its inhibition increases axonal density at lesion borders, underscoring its potential therapeutic significance (Malvandi et al. 2022; Han et al. 2023).

miR‐146a, which is highly enriched in glial cells, plays a critical role in regulating inflammation and cell differentiation. It targets superoxide dismutase 2 (SOD2), thereby modulating oxidative stress responses, and inhibits inflammatory mediators such as IRAK1, IRAK2, TRAF6, IL‐6, and TNF‐α in astrocytes. By regulating astrocyte‐associated transcripts, including Nlgn1, Nova1, and Syt1, miR‐146a promotes the differentiation of NSCs toward astrocytes while limiting proneuronal fate. Thymosin β4 (Tβ4)–induced miR‐146a enhances OPC differentiation by reducing ERK/JNK1 activation and c‐Jun‐mediated repression of myelin basic protein (MBP). Through these mechanisms, miR‐146a integrates inflammatory regulation with OPC maturation and remyelination (Qiu et al. 2024; Iyer et al. 2012). Among pro‐differentiation miRNAs, miR‐219 is recognized as a key regulator of OL lineage development. miR‐219, which is highly expressed during maturation, promotes differentiation by suppressing inhibitors such as FoxJ3, ZFP238, Sox6, Hes5, and Etv5. It also restricts OPC proliferation by targeting PDGFRα and facilitates remyelination by inhibiting Lingo1. In vivo studies demonstrate that administration of miR‐219 agomiR after a C5 contusion injury enhances OPC differentiation, OL numbers, myelin repair, and motor recovery. Similarly, transplantation of miR‐219–overexpressing OPCs after T12 compression injury further improves white matter preservation, axonal regeneration, and functional recovery (Qiu et al. 2024; Nazari et al. 2021). miR‐338 acts in concert with miR‐219 by targeting transcriptional inhibitors such as Sox6 and Hes5. Simultaneous deletion of both miRNAs results in severe demyelination, underscoring their synergistic role in myelin formation. In contrast, miR‐138 supports early OPC differentiation by inhibiting Sox4, but may delay later stages of maturation. This distinguishes miR‐138 from miR‐219, which functions across developmental stages (Qiu et al. 2024). miR‐23 regulates OL differentiation and myelin production. Overexpression of miR‐23 enhances myelin formation by targeting PTEN and activating the Akt/mTOR signaling pathway. Additionally, miR‐23a suppresses lamin B1 (LMNB1), a known negative regulator of OL maturation (Lin and Fu 2009). miR‐23a is overexpressed in multiple sclerosis (MS), where it improves OL differentiation and myelination, highlighting its potential therapeutic value for promoting remyelination (Qiu et al. 2024; Ma et al. 2014). The let‐7 family of miRNAs is highly expressed during peripheral nervous system myelination and exhibits an inverse relationship with Lin28B expression. Persistent Lin28B expression reduces let‐7 levels and disrupts Schwann cell myelination. Let‐7 enhances myelination by upregulating Krox20 (Egr2) through inhibition of the Notch signaling pathway. Although let‐7 is also abundantly expressed in the CNS, its essential role in CNS myelination remains incompletely understood (Gökbuget et al. 2015; Li et al. 2016).

Several miRNAs contribute to axonal regeneration and remyelination (Table 4). miR‐133b promotes neurite outgrowth by suppressing RhoA and reducing CSPG‐mediated inhibition through activation of ERK1/2 and PI3K/Akt signaling pathways (Lu et al. 2015). miR‐132 facilitates axon extension by targeting Rasa1 and regulating neuronal morphogenesis via CREB (Hancock et al. 2014). miR‐431 enhances axonal growth by silencing Kremen1 and modulating neurite length through chondrolectin and Kif3B (Wu and Murashov 2013). Additionally, miR‐124 supports neurite elongation by suppressing ROCK1, KLFs, and STAT3, while a miR‐138/SIRT1 feedback loop further promotes axon repair (Li et al. 2016).

**TABLE 4:** MiRNAs regulating axon regeneration and remyelination after SCI.

MiRNA	Expression after SCI	Major target(s)	Biological significance	Ref
miR‐7a	Not determined (no SCI model)	Pax6, NeuroD4, CNPase, Sp1	Promoted OPC specification and proliferation.	(Qiu et al. 2024; Zhao et al. 2012)
miR‐124	Time‐dependent (early ↑, late ↓)	ROCK1, KLFs, STAT3	Promoted neurite elongation and axonal regeneration.	(Li et al. 2016)
miR‐125a‐3p	↑ (MS/EAE models)	Slc8a3, Gas7, Fyn, Nrg1, RhoA	Inhibited oligodendrocyte maturation and remyelination.	(Lecca et al. 2016; Marangon et al. 2020; Qiu et al. 2024)
miR‐132	Not determined (no SCI model)	Rasa1 (Ras signaling)	Promoted axon extension via Rasa1 repression.	(Hancock et al. 2014)
miR‐133b	Not determined (no SCI model)	RhoA; ERK1/2 and PI3K/Akt signaling	Promoted neurite outgrowth and axonal regeneration.	(Lu et al. 2015)
miR‐138	↑ During OPC differentiation	Sox4	Promoted early oligodendrocyte differentiation.	(Potzner et al. 2007; Dugas et al. 2010; Yeh et al. 2013; Qiu et al. 2024)
miR‐146a	↑ During OPC differentiation	ERK1, JNK1, c‐Jun	Promoted OPC differentiation via c‐Jun inhibition.	(Ammal Kaidery et al. 2021)
miR‐184	↑ During OPC differentiation and OL maturation	Sox1, BCL2L1, Lingo1	Promoted oligodendrocyte differentiation and myelination.	(Afrang et al. 2019; Qiu et al. 2024)
miR‐219	↑ During OPC differentiation	PDGFRα, FoxJ3, Sox6, Hes5, Etv5, Lingo1	Promoted OPC differentiation and remyelination.	(Nazari et al. 2021; Wang et al. 2017; Dugas et al. 2010; Li et al. 2019)
miR‐338	↑ During OPC differentiation	Sox6, Hes5	Promoted OPC differentiation and myelination.	(Dugas et al. 2010; Wang et al. 2017; Qiu et al. 2024)
miR‐431	Not reported (upregulated after sciatic nerve injury)	Kremen1; Wnt/β‐catenin signaling	Promoted axon regeneration via Kremen1 repression.	(Wu and Murashov 2013)

miR‐184 promotes commitment to the OL lineage. Its expression increases during OPC differentiation, and overexpression in neural progenitor cells directs these cells toward an OL fate by suppressing Sox1 and BCL2L1. miR‐184 also inhibits Lingo1, thereby facilitating the myelination process (Table 4) (Afrang et al. 2019). In contrast, several miRNAs inhibit OPC differentiation. For example, miR‐26b exacerbates motor impairment after SCI by targeting adrenomedullin (ADM) and hindering OPC maturation (Cheng et al. 2020). Similarly, miR‐24 suppresses OPC differentiation and increases inflammatory signaling by downregulating ADM and MBP (Lei et al. 2020). miR‐212, which is reduced following SCI, negatively regulates OL maturation. Overexpression of miR‐212 reduces the expression of CNPase, PLP1, and MBP, suggesting that decreased miR‐212 after injury may enhance remyelination (Figure 2) (Wang et al. 2017). miR‐7a maintains OPCs in an immature state by targeting neuronal differentiation regulators such as Pax6 and NeuroD4, as well as myelin‐related genes including CNPase and Sp1 (Zhao et al. 2012). miR‐9 levels decrease during the transition from OPCs to mature OLs and regulate genes such as PMP22 and serum response factor (SRF). In demyelinating conditions, miR‐27a and miR‐125a‐3p are upregulated. miR‐27a disrupts OPC proliferation and differentiation by activating Wnt/β‐catenin signaling through inhibition of adenomatous polyposis coli (APC), ultimately leading to decreased myelin thickness (Qiu et al. 2024; Tripathi et al. 2019; Galloway and Moore 2016). miR‐125a‐3p impairs OL differentiation by targeting genes such as Slc8a3 and Gas7, as well as key regulators of myelination including Fyn, neuregulin 1 (Nrg1), and RhoA. Silencing miR‐125a‐3p has been shown to enhance remyelination (Table 4) (Lecca et al. 2016; Marangon et al. 2020).

A number of miRNAs regulate OL development. For example, miR‐199a‐5p targets C11Orf9, a gene essential for OL maturation. Inhibition of miR‐199a‐5p restores expression of the myelin regulatory factor (MRF) and improves functional outcomes in neurotoxicity models (Letzen et al. 2010; Zhang et al. 2021). miR‐214, miR‐145‐5p, and miR‐142a also function as negative regulators of differentiation by targeting MRF. In contrast, the miR‐17–92 cluster promotes OPC proliferation by suppressing PTEN and activating the Akt signaling pathway. miR‐297c‐5p inhibits cell‐cycle progression by suppressing CCNT2, thereby increasing the number of mature OLs. Additionally, miR‐146b‐5p supports OPC survival and reduces oxidative stress by downregulating Brd4 and activating antioxidant pathways, which ultimately promotes remyelination following injury (Qiu et al. 2024).

## 5 SCI Therapy Based on MiRNAs

Current therapeutic strategies seek to restore miRNA homeostasis by either replacing downregulated miRNAs with synthetic mimics or inhibiting aberrantly expressed miRNAs using antagomirs, locked nucleic acids (LNAs), miRNA sponges, or antisense oligonucleotides. Although these approaches have demonstrated promising efficacy in preclinical studies, their clinical translation is hindered by poor nuclease stability, short circulating half‐life, off‐target effects, potential immunogenicity, and inefficient cellular uptake. Furthermore, rapid degradation by circulating RNases and limited penetration across the BSCB significantly reduce therapeutic efficiency. These challenges underscore the necessity for advanced delivery systems that enhance miRNA stability and facilitate targeted delivery to the spinal cord (Bader et al. 2010; Momin et al. 2021; Dasgupta and Chatterjee 2021; Yang 2015).

Modulation of dysregulated miRNAs has demonstrated considerable therapeutic potential in SCI by reducing secondary injury and facilitating neurological recovery. Administration of miRNA mimics, such as miR‐20a, miR‐21, miR‐23b, miR‐27a, miR‐124, miR‐126, miR‐133b, miR‐199, miR‐210, miR‐320a, miR‐494, and miR‐497, has been associated with enhanced angiogenesis, maintenance of BSCB integrity, decreased lesion size, and improved functional outcomes. Conversely, inhibition of pathogenic miRNAs, including miR‐20a, miR‐486, miR‐223, and miR‐320, yields neuroprotective effects and enhances locomotor function in experimental SCI models. Additionally, combinatorial strategies, such as bone marrow stromal cell transplantation combined with adeno‐associated virus (AAV)‐mediated miR‐383 delivery, have demonstrated superior neuroprotective outcomes compared to single‐treatment modalities (Deng and Chen 2023; Ho et al. 2022; Zhang et al. 2023; Sun et al. 2018).

Several delivery platforms have been developed to overcome these challenges (Table 5) (Islam and Tom 2022; Assinck et al. 2017; Hou et al. 2021; Eygeris et al. 2022; Moosavi et al. 2025; Mitragotri et al. 2017; Teleanu et al. 2019; Kalluri and LeBleu 2020; Pegtel and Gould 2019; Chen et al. 2022; Ju et al. 2025; Poongodi et al. 2025; Wang et al. 2023; Hasan et al. 2025; Deng et al. 2026). Viral vectors enable sustained gene expression but are constrained by immunogenicity and safety concerns. Lipid and polymeric nanoparticles enhance miRNA stability and controlled release, although further optimization is necessary for efficient spinal cord targeting. Exosomes demonstrate strong BSCB penetration and low immunogenicity. Stem cell–mediated delivery offers both regenerative capacity and sustained miRNA transport. Intrathecal administration bypasses the BSCB and achieves high local bioavailability, but it remains invasive. No single delivery strategy is universally optimal for SCI; therefore, selection should be guided by the therapeutic objective, target cell population, stage of injury, and route of administration.

**TABLE 5:** Comparison of current miRNA delivery strategies for SCI therapy.

Delivery strategy	Advantages	Limitations	SCI‐specific considerations	Clinical status	Ref
Viral vectors (AAV/lentivirus)	High transduction efficiency; sustained gene expression	Immunogenicity, limited cargo capacity (AAV), insertional mutagenesis (lentivirus)	Enable long‐term gene modulation but require optimized vector, promoter, and delivery route for efficient spinal cord targeting.	Preclinical	(Islam and Tom 2022; Assinck et al. 2017)
Lipid nanoparticles	Protect RNA cargo; scalable production	Limited intrinsic CNS/spinal targeting	Require ligand modification or BBB/BSCB‐opening strategies for efficient spinal cord delivery.	Preclinical/early translational	(Hou et al. 2021; Eygeris et al. 2022; Moosavi et al. 2025)
Polymeric nanoparticles	Controlled release	Potential cytotoxicity	Improve local retention and sustained miRNA release at the injury site.	Preclinical	(Mitragotri et al. 2017; Teleanu et al. 2019)
Exosomes	Natural carrier; low immunogenicity	Low yield; isolation and purification challenges	Efficient BSCB penetration and intrinsic targeting of injured spinal tissue.	Preclinical	(Kalluri and LeBleu 2020; Pegtel and Gould 2019; Chen et al. 2022; Ju et al. 2025)
Stem cell–mediated delivery	Cell homing; regenerative potential	Manufacturing complexity; variability; potential tumorigenicity	Combines tissue regeneration with sustained miRNA delivery and immunomodulation.	Experimental	(Assinck et al. 2017; Poongodi et al. 2025; Ju et al. 2025)
Intrathecal administration	Direct delivery to the spinal cord with high local bioavailability	Invasive; repeated dosing may require specialized procedures	Bypasses the BSCB.	Preclinical; clinically feasible via lumbar intrathecal injection	(Wang et al. 2023; Hasan et al. 2025; Deng et al. 2026)

A critical factor in miRNA‐based therapy is the temporal regulation and context‐dependent expression of miRNAs following SCI. The biological functions of many miRNAs are highly dependent on the stage of injury, suggesting that therapeutic efficacy is determined not only by the specific miRNA selected but also by the timing of its administration. For instance, miR‐21 demonstrated neuroprotective and antiapoptotic effects during the acute phase of SCI by suppressing PTEN and PDCD4, which promoted neuronal survival and reduced secondary injury. In contrast, prolonged miR‐21 expression in later stages can increase astrocyte activation and ECM remodeling, leading to glial scar formation and restricting axonal regeneration. Comparable context‐dependent effects have been observed for other miRNAs involved in inflammation and tissue repair. Consequently, future miRNA‐based therapeutic strategies should account for the temporal dynamics of miRNA expression and implement stage‐specific and cell‐specific modulation to optimize neuroprotection while minimizing negative impacts on chronic tissue remodeling. Administration of the same miRNA at different stages of SCI may result in distinct, and sometimes opposing, biological outcomes.

## 6 Challenges and Future Perspectives

MiRNAs regulate numerous injury‐related processes, including apoptosis, inflammation, redox homeostasis, vascular integrity, demyelination, and reactive gliosis. They represent promising therapeutic targets due to their ability to modulate broad signaling pathways. For example, miR‐21 influences apoptosis, inflammation, astrocyte behavior, and ECM remodeling. In contrast, miR‐223 promotes neuronal survival and modulates leukocyte‐mediated inflammation and angiogenic signaling. miR‐124 reduces secondary degeneration by directing microglia toward an anti‐inflammatory phenotype, whereas miR‐219 facilitates OL differentiation and myelin repair, thereby supporting structural stability. Additionally, several miRNAs have emerged as potential diagnostic and prognostic biomarkers for SCI; however, their clinical reliability is constrained by variability in injury severity, sampling time, and analytical methodologies.

Although preclinical studies have demonstrated promise, clinical translation remains limited due to delivery challenges such as inadequate tissue penetration, rapid molecular degradation, insufficient cell‐specific targeting, and the potential for off‐target or immune‐mediated adverse effects. Additionally, the complex and interconnected nature of SCI pathology suggests that modulation of a single miRNA may be inadequate for achieving sustained functional recovery, thereby underscoring the potential benefits of combinatorial therapeutic strategies. Advances in viral vectors, nanoparticle systems, exosome carriers, and stem cell platforms are enhancing targeting precision and long‐term efficacy. Ongoing research into miRNA expression profiles, biomarker validation, and integrated neuroprotective and regenerative approaches will be critical for the development of future therapies.

## Author Contributions

**Saeed Vafaei‐nezhad**: conceptualization, supervision. **Seyyedeh Fahimeh Talebi**: investigation, writing – original draft, data curation. **Hossein Kalarestaghi**: writing – original draft, data curation, investigation. **Ehsan Sheikh Hesabi**: writing – original draft, writing – review and editing, software.

## Funding

The authors have nothing to report.

## Ethics Statement

The authors have nothing to report.

## Conflicts of Interest

The authors declare no conflicts of interest.

## Data Availability

Data sharing is not applicable to this article, as no new datasets were generated or analyzed during the current study.
